# A perspective on the diagnosis of cracked tooth: imaging modalities evolve to AI-based analysis

**DOI:** 10.1186/s12938-022-01008-4

**Published:** 2022-06-15

**Authors:** Juncheng Guo, Yuyan Wu, Lizhi Chen, Shangbin Long, Daqi Chen, Haibing Ouyang, Chunliang Zhang, Yadong Tang, Wenlong Wang

**Affiliations:** 1 School of Mechanical and Electrical Engineering, Guangzhou University, Guangzhou, 510006 China; 2 School of Biomedical and Pharmaceutical Sciences, Guangdong University of Technology, Guangzhou, 510006 China

**Keywords:** Review of oral diagnosis, Image processing, Artificial intelligence, Survey of crack detection

## Abstract

Despite numerous clinical trials and pre-clinical developments, the diagnosis of cracked tooth, especially in the early stages, remains a challenge. Cracked tooth syndrome is often accompanied by dramatic painful responses from occlusion and temperature stimulation, which has become one of the leading causes for tooth loss in adults. Current clinical diagnostical approaches for cracked tooth have been widely investigated based on X-rays, optical light, ultrasound wave, etc. Advances in artificial intelligence (AI) development have unlocked the possibility of detecting the crack in a more intellectual and automotive way. This may lead to the possibility of further enhancement of the diagnostic accuracy for cracked tooth disease. In this review, various medical imaging technologies for diagnosing cracked tooth are overviewed. In particular, the imaging modality, effect and the advantages of each diagnostic technique are discussed. What’s more, AI-based crack detection and classification methods, especially the convolutional neural network (CNN)-based algorithms, including image classification (AlexNet), object detection (YOLO, Faster-RCNN), semantic segmentation (U-Net, Segnet) are comprehensively reviewed. Finally, the future perspectives and challenges in the diagnosis of the cracked tooth are lighted.

## Introduction

Crack detection for hard tissue, especially for oral cracked tooth may be considered as one of the typical combinational problems of oral medicine and engineering structure monitoring. In 1964, Cameron first introduced the concept of cracked tooth symptom, which was initially described as the incomplete fracture of the anterior molar [1]. Later on, the American Association of Endodontists classified cracked tooth symptom into more detailed categories based on four main features: the origin of the cracked tooth, the trend of the crack, the clinical symptoms, and the pulpal activity [2–4]. The cracked tooth was mainly caused by excessive occlusal forces and iatrogenic causes (e.g., the use of dental rotary instruments during cavity preparation) [5]. Early cracked tooth symptom was extremely easy to be misdiagnosed due to the tiny cracks and its unclear clinical response. If these cracks were not detected and treated in time, the cracks would gradually deepen to dentin layers as the affected tooth continues to be stressed while chewing, which may induce pulpitis and even cause a complete fracture of the tooth [6]. Nowadays, cracked tooth symptom has become one of the major causes of tooth loss in adults [7]. The clinical study of cracked tooth, particularly the diagnosis of the cracked tooth (namely, crack detection for hard tissue) has attracted significant attention and interest in human oral health and engineering structure condition monitoring.

Currently, the diagnosis of cracked tooth is mainly based on clinical symptoms [8]. For the suspected teeth, the clinician could determine them by several traditional clinical tests, such as occlusion test method [9], probing method [10], staining method [11], cold stimulation method [12] and light transillumination method [13]. However, some limitations may exist in these methods. For example, the cold stimulation method is not accurate enough because the sense of pain is not obvious in the early stages of cracked tooth [12]. The staining method should be performed with a surgical microscope, which lacks convenience [11]. The probing method can cause a lot of pain to the patient during clinical testing due to improper operation [10]. The occlusion test may aggravate the disease and even cause the tooth to fracture because of the increased local stress [14]. The Light transillumination method cannot distinguish the type and depth of the hidden cracks [13]. Besides, the early clinical symptoms of cracked tooth can be easily confused with other diseases, such as pulpitis, periodontal disease and periapical infection [15]. Moreover, not all cracked teeth have visible cracks or other symptoms. The difficulty in detecting cracked tooth lies in the diagnosis of an asymptomatic cracked tooth, because their insidious nature makes it very challenging for clinicians, especially young doctors. On the other hand, it can be inaccurate due to the doctor's visual fatigue or misjudgment.

X-ray-based modern crack detection methods have been widely developed in recent years, which brought great benefits to the diagnosis of cracked tooth in clinical practice. Whereas, the diagnosis of the cracked tooth symptom strongly depends on the experience of the clinicians. Misdiagnosis and related wrong therapy may happen simply based on the inspection by the dentists. Modern image treatment methods especially advanced artificial intelligence (AI) algorithms (such as convolutional neural networks(CNN), U-net) have strong feature extraction and generalization capabilities [16–20], which may further help to increase the efficiency and the accuracy of the diagnosis [21]. For example, Cernazanu-Glavan et al. [18] used CNN to achieve segmentation of bone tissue from X-ray images without human intervention. Dhungel et al. [19] proposed an automatic method for the segmentation of mass in mammograms based on CNN. In recent years, AI with deep learning as the core has developed rapidly. Ronneberger et al. [20] proposed a magnificent U-net network with much fewer training data sets, which could apply to various biomedical segmentation problems. The key tasks in computer-aided diagnosis normally relate to the treatment of medical images. Nowadays, CNN, as one of the representative AI technologies, has achieved great success, particularly in the field of image segmentation. Therefore, the diagnostic method improving with deep learning algorithms may be considered as one of the future research directions.

Current clinical diagnostical approaches for cracked tooth have been widely investigated based on X-rays, optical light, ultrasound wave, etc. However, a symmetrical summary concerning each methodology and the possible image treatment methods of crack detection seems rarely reviewed. In this review, various medical imaging techniques in detecting fractures for cracked tooth were extensively summarized and discussed concerning methods, such as computer tomography (CT), cone-beam computed tomography (CBCT), ultrasound, micro-computed tomography (micro-CT), optical coherence tomography (OCT) and magnetic resonance imaging (MRI). And then, AI-based image analysis methods aimed at crack segmentation and recognition were also reviewed and briefly discussed. This paper aims to help conduct more targeted research and provide useful assistance in the development of modern clinical methods for the detection and diagnosis of cracked tooth and other similar crack disease in medical or related engineering structure.

## Imaging modalities in the diagnosis of cracked tooth

**Fig. 1:**
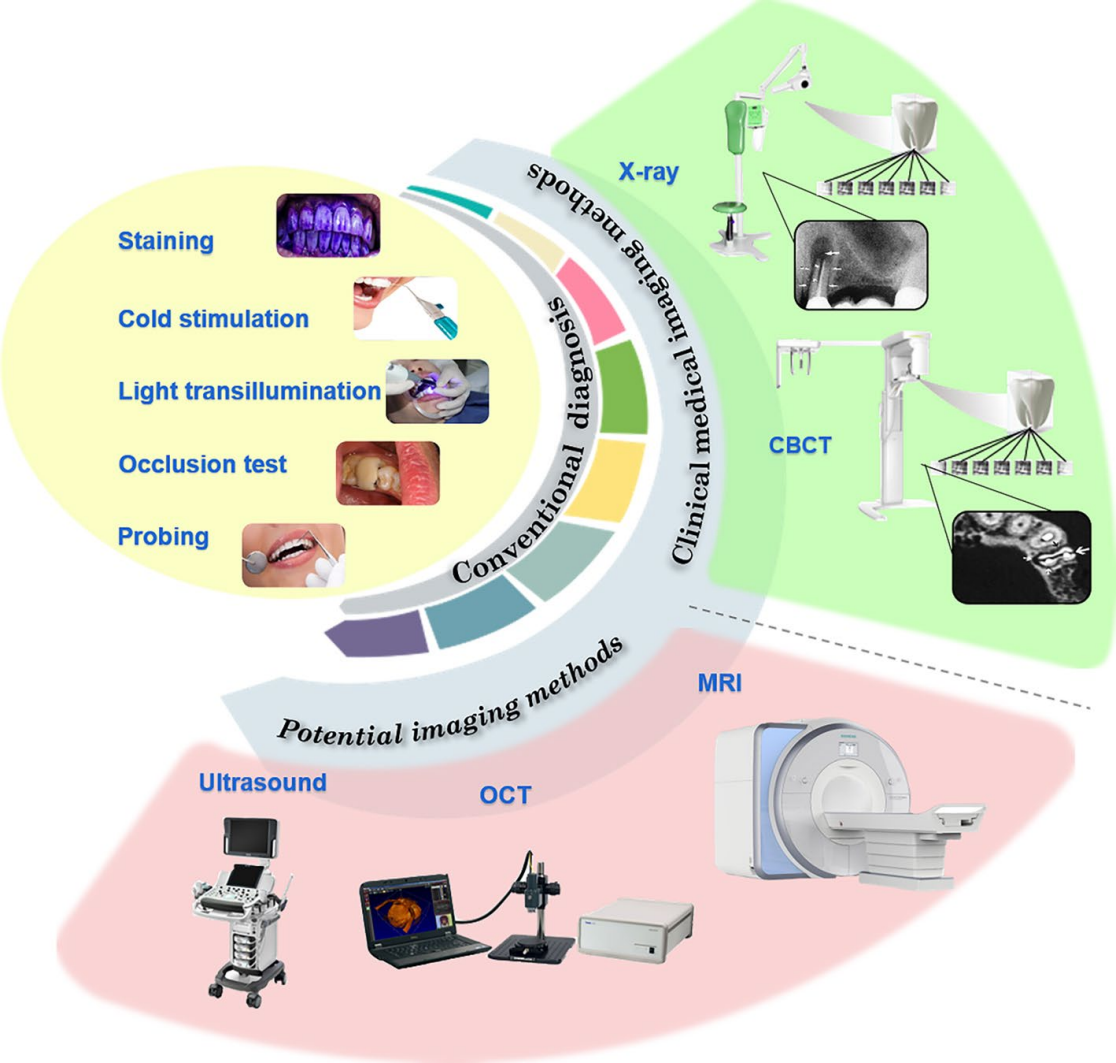
Illustrations of the methods for the diagnosis of cracked tooth

Medical imaging plays a vital role in the detection of microcracks in teeth. Depending on the types of imaging sources, this section has described different medical imaging-based techniques for the crack detection of hard tissue (as illustrated in Fig. 1).

### Detection of cracked teeth based on X-rays

The applications of X-rays have made a significant leap for clinical diagnosis. The following section describes X-ray-based techniques used for oral detection, including oral X-rays, CBCT and micro-CT.

#### Oral X-ray radiograph

**Fig. 2:**
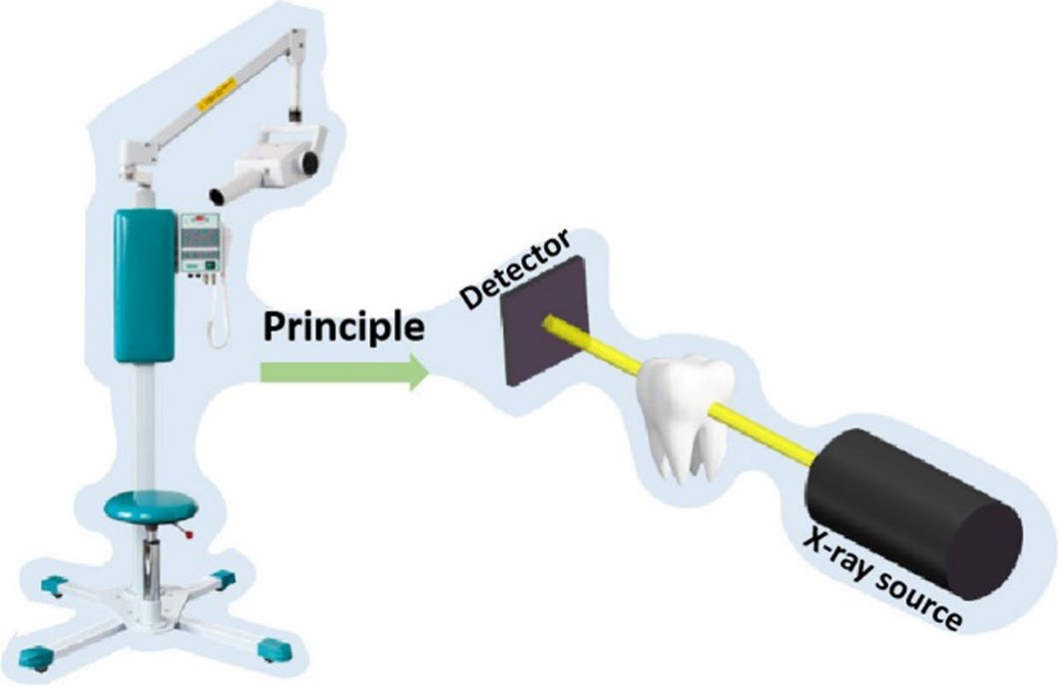
Illustration of the measurement of oral X-ray radiograph

As shown in Fig. 2, radiographs based on X-rays enable to visualize different components inside the tissue according to the distinct degrees of the ray absorption [22]. In early time, oral X-ray radiograph was severed as a common technique to determine the presence of periapical disease. It may provide extensive details in the oral cavity and allow the dentists to locate the tooth cracks [23]. In vitro detection of root fractures had demonstrated the validity of the diagnosis for the cracked tooth both with digital radiography and conventional film X-rays [24], but the resolution and the imaging effect were strongly influenced by the angle of incidence of the beam rays [24]. Particularly, fractures would be misdiagnosed if the X-ray beam did not pass through the fracture line [25]. Planar periapical intraoral radiograph may be useful in some cases. Nevertheless, due to the superimposition of anatomic structures onto features of diagnostic interest, missed diagnosis may still occur when dealing with the case, such as relatively non-displaced fracture [26].

Diagnosis of the vertical root fractures (VRF) was probably one of the challenging tasks for 2D oral X-ray radiograph [27–29]. VRF is a type of cracked tooth, which is defined as a fracture that originates from the coronal (enamel) or apical (root) portion of the tooth and usually extends faciolingually [30]. The longitudinal cracks orientated in the buccolingual direction may lead the beam to be strongly disturbed by the adjacent tissues, which may reduce the imaging quality of the detection. Besides, information concerning the trend of the crack propagation or the crack orientation could hardly be obtained from 2D images generated by oral radiograph [31, 32]. Consequently, three-dimensional imaging diagnostic system may be a better choice for the detection and quantitative evaluation of the crack with relative complex geometry and orientation (i.e., VRF).

#### CBCT

The meaningful and commercialized 3D medical imaging was achieved by the rapid development of computed tomography, which was an integrated image acquisition technique combining the ideas of X-ray fractionated transmission, cross-sectional scanning, and secondary image treatment of 3D reconstruction [33, 34]. Compared with oral dental radiography, a clinical study [35] of 42 suspected cases in 47 patients indicated that tooth fracture especially VRF detected by CT showed significantly higher diagnostic accuracy. However, the usage of conventional CT was limited due to its high radiation dose, relatively low spatial resolution, large volume and high cost [34, 36].

**Fig. 3:**
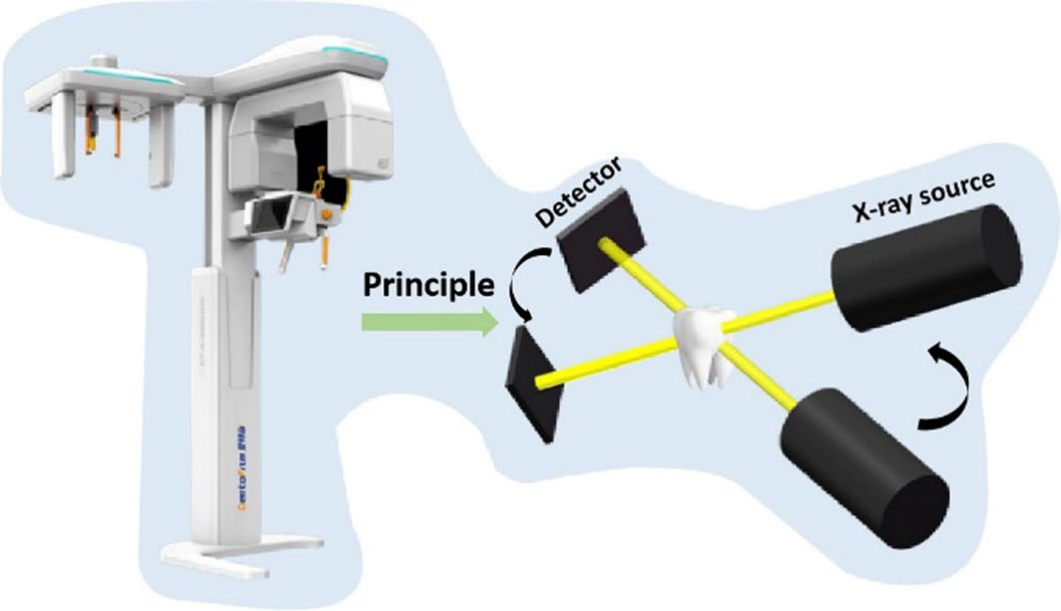
Illustration of the measurement of CBCT

Later on, cone-beam technology was adapted for dental applications [37]. As the most commonly used scanning technique in dental clinics nowadays, CBCT reduces radiation dose through locally circumferential projection around the mouth [38, 39]. With the improvements of scanning modes (from 2D sector beam scanning to cone-beam scanning) and detectors (from linear detector to panel detector), the ability of multi-tissue reconstruction and multi-angle visualization significantly improved the imaging resolution, and meanwhile, reduced the interference to the CT imaging due to metal dental implants [40, 41]. The measurement is illustrated in Fig. 3.

CBCT has been proven to be a valuable tool in modern dental clinical diagnosis [42, 43]. Diagnosis with CBCT offers a 3D reconstruction of the tissue and provides a relatively high accuracy [43–46], especially for the case of detecting vertical root fractures and periapical lesions compared to oral X-ray radiograph [47]. A Clinical study conducted by Lin et al. [48] has shown that CBCT can simultaneously visualize the anatomical structure of the teeth, the morphology of the root canal and the periodontal tissue. Kalyan et al. [49] performed multi-layer sequential scans on the affected tooth, which indicated the location of the crack, its direction and the relationship between the apical part and the pulp of the tooth. Furthermore, as listed in Table 1, various studies have been conducted to compare CBCT and periapical radiography (PR) in the diagnosis of cracked tooth. Among them, it may be concluded that CBCT achieved higher sensitivity and accuracy in the diagnosis of root fracture [50]. The overall specificity of both PR and CBCT was comparable, and the clinical study indicated that the two diagnostic modalities might not be significantly affected by root canal fillings in endodontically treated teeth [51]. What’s more, CBCT was probably more susceptible due to streaking artifacts caused by the radiopaque root fillings [52].

**Table 1:** Comparative study between CBCT and PR (periapical radiography)

Literature	Method	Subjects	Results (%)				
			ACC	SEN	SPE	PPV	NPV
[145]	CBCT	Dogs’ anterior maxillae		82	90	86	
	PR			87	95	92	
[146]	CBCT	Human mandibular premolar and molar teeth		98	100	99	99
	PR			65	100	100	71
[52]	CBCT	Human teeth with gutta-percha		68.8	36.7	27.7	75
	PR			19.2	97.5	61.2	78
[147]	CBCT	Human teeth (40 premolars and 40 molars)	86	77.5	91.3		
	PR		66	37.5	95		
[35]	CBCT	Human teeth		70	100	100	64
	PR			23	100	100	100
[50]	CBCT	135 human teeth (49were endodontically treated)	91.9	89.5	97.5	98.8	79.6
	PR		48.1	26.3	100	100	36.4

PPV: Positive Predictive Value; NPV: Negative Predictive Value; SEN: Sensitivity; SPE: Specificity; ACC: Accuracy

The voxel size of the CBCT was reported to be around 75–400 μm [51], which made the diagnosis of the finer micro-crack impossible [53]. Although smaller voxel size of the CBCT could be achieved by increasing the radiant intensity, the associated side-effect was undesirable [54, 55]. Therefore, to keep the radiation dose as low as possible while maintaining high image quality, Senem et al. [32] compared the diagnostic accuracy of CBCT scans with different voxel resolutions, which suggested that a resolution of 0.2 mm may be the appropriate choice for clinical diagnosis.

Overall, CBCT has been considered as one of the main approaches for oral clinical diagnosis. However, some challenges remain: (a) as mentioned above, although CBCT may reduce imaging artifacts induced by radiopaque materials, the resulting noise and interference are unavoidable [26, 56, 57]; (b) limited resolution may obscure micro cracks [58–61], particularly for the early time of the crack tooth symptom.

#### Micro-computed tomography

Micro-computed tomography (abbreviated as micro-CT or μCT) has provided a breakthrough in diagnostic medical imaging. Micro-CT technology utilizes a microfocus X-ray source to circumferentially illuminate the sample, allowing three-dimensional imaging of the tooth structure [62, 63]. The microfocus spot X-ray sources and high-resolution detectors of the micro-CT system can achieve a higher sensitivity compared to CBCT [64]. By adjusting the scanning parameters, the resolution of micro-CT could reach up to 9 μm, which effectively realized the early detection of VRF [65]. In addition, micro-CT was also served as the standard for verifying apical dentinal micro-fractures after root canal treatment [66]. However, the high resolution of the micro-CT brings some problems. For example, the imaging is quite prone to artifacts, which may cause image aliasing and affect the quality [67]. Besides, the ultrahigh resolution of micro-CT is usually accompanied by a radiation dose up to 1500 mGy, which significantly exceeds the clinical CT reference range of 1 to 70 mGy [68]. Thereby, if micro-CT was applied in the clinical diagnosis, the clinician should carefully balance the contradiction between the imaging resolution and radiation exposure.

### Other imaging techniques in cracked tooth diagnosis

In addition to X-ray-based methods, other imaging modalities, such as ultrasound, magnetic resonance imaging (MRI) and optical coherence tomography (OCT) have also been developed because of the non-radiative characteristics. In the following sub-sections, these imaging techniques are briefly reviewed and discussed.

#### Ultrasound

Ultrasound is a mechanical vibration wave whose frequency is higher than 20 kHz, which is beyond the upper limit of the human's hearing [69]. In the 1960s, LEE et al. [70] started the work on ultrasonic detection of dental hard tissue. Since then, many researchers have conducted ultrasonic imaging experiments on isolated teeth and measured the structural dimensions of the tissues, i.e., the thickness of enamel and dentin [71–73].

Because of the radiation-free and non-invasive characteristics, ultrasound detection technology showed significant advantages in the field of oral diagnosis, for example, detection of caries [74], periapical lesions [75] and dental fracture and cracks [76]. Culjat et al. [76] have shown the ultrasonic imaging could clearly distinguish the cracked and uncracked parts of the artificially made cracked tooth (microcrack width: 25 μm). Singh et al. [77] conducted a comparative study by the ultrasound imaging system to compare the ability to detect the cracks within gold, amalgam and porcelain restorations, which indicated the accuracy of ultrasound imaging might be influenced by gold fillings. Furthermore, by combining the ideas of ultrasonic vibration and infrared imaging, ultrasonic vibration infrared thermography, also named vibrothermography (VibroIR) was developed to detect microcracks based on the frictional heat generated by ultrasonic vibrations. The work from Matsushita et al. [78] indicated the hidden fissures in dental tissues ranging from 4 to 35.5 μm could be captured by VibroIR. More recently, dual-contrast photoacoustic tomography combining ultrasound was reported to be applied for the detection of dental lesions at an early stage, which indicated a reasonable spatial resolution and optical contrasts for deep tissue imaging [79].

It should be noted that the in-vitro cracked tooth model was usually constructed with similar acoustic characteristics to natural tooth enamel and dentin, but the complex geometry of the tooth and the unclear mechanism of ultrasound on dentin and enamel may also induce some difficulties when analysing the signals of ultrasonic imaging. Besides, specific knowledge of ultrasonic inspection may be necessary for the diagnosis of oral cracked tooth, which also reduces the feasibility of the method. That may be the reason why no more related literature could be found concerning the performance of ultrasonic detection in the further clinical diagnosis of the oral cracked tooth.

#### OCT

**Fig. 4:**
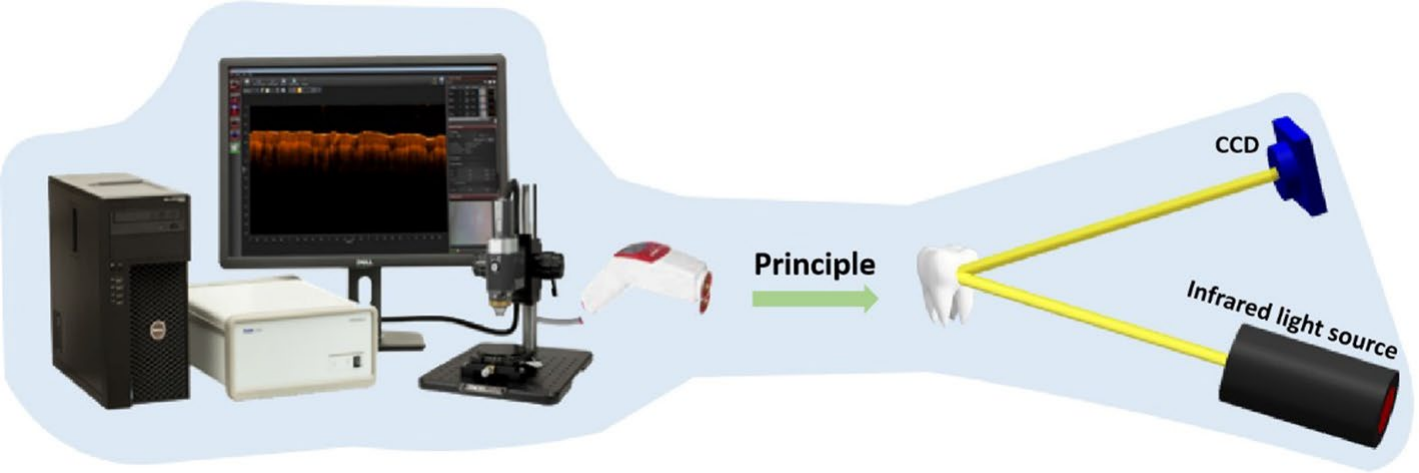
Illustration of the measurement of OCT

Optical coherence tomography (OCT) was first proposed by Huang et al. [80] as a non-invasive, high-resolution, zero-radiation optical imaging technique, which has been widely used in biomedical applications recently, as shown in Fig. 4. Based on the interference of the weak coherent light, OCT could achieve two-dimensional or three-dimensional imaging of biological tissues by detecting the backward reflection or several scattering signals resulted from incident coherent light focused at different depths of the biological tissues. At present, OCT has been applied in the oral cavity as one of the auxiliary diagnostic approaches for cases, such as enamel surface demineralization [81], early caries damage [82], hidden tooth fracture [83], resin filling microleakage [84] and dentinal fracture after root canal treatment [85].

**Fig. 5:**
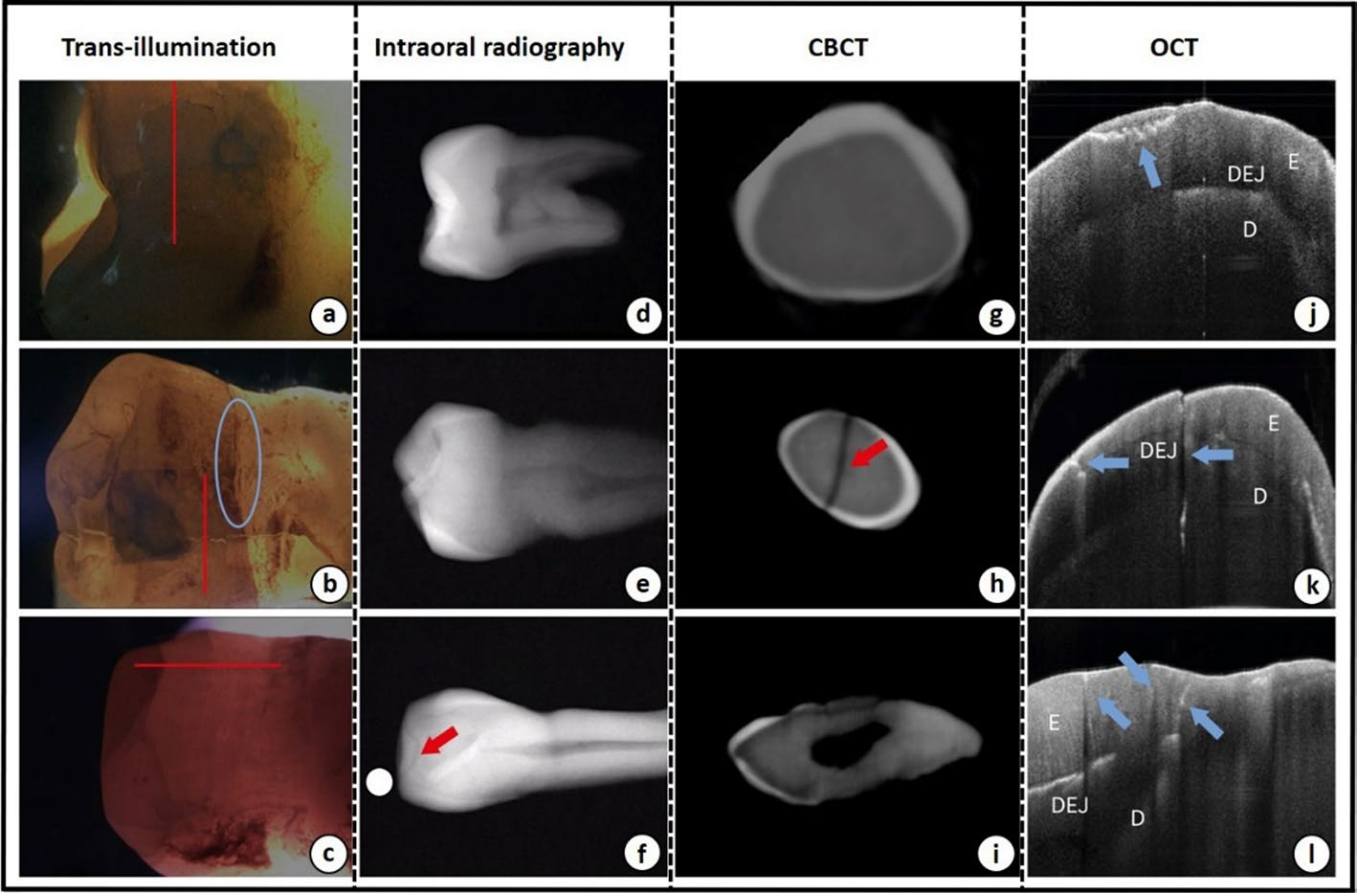
Comparison study of four crack detection methods: (**a**–**c**) images from trans-illumination detection; (**d**–**f**) images from intraoral radiography; (**g**–**i**) images from CBCT; (**j**–**l**) images from OCT. The red line indicates the cross section of CBCT and the OCT scan line. Red and blue arrows indicate crack lines. The blue circle indicates a false-positive crack in trans-illumination detection. The image resource was cited from reference [53]. Copyright © 2017. Korean Academy of Periodontology publishing

For the observation of dental fissures, the resolution of OCT can reach 10 μm, which is comparable to micro-CT. Research conducted by Shemesh et al. [86] revealed that the sensitivity and specificity of OCT for the diagnosis of vertical root fractures were significantly higher than 90%. Comparable studies from Kim et al. [53] have also been carried out between OCT, intraoral radiography, CBCT and trans-illumination images, which indicated that images obtained from OCT have the highest resolution for detecting micro-cracks [53]. As shown in Fig. 5, for three selected diagnostic areas, OCT could clearly capture the tooth components (enamel and dentin) associated with multiple fissures inside the tissue, whereas, not all the micro-cracks could be visualized by the X-ray based methods. Furthermore, with the advent of high-speed swept-frequency light sources, swept-source optical coherence tomography (SS-OCT) has been developed with an axial resolution of 5.3 µm and an axial scan rate of 100,000 scans per second [87]. Studies of the diagnosis for natural root fractures in isolated molars have shown that SS-OCT possessed higher sensitivity and specificity compared to micro-CT [88, 89].

#### MRI

Another important non-invasive medical diagnosis method is magnetic resonance imaging (MRI), which uses non-ionizing radiofrequency electromagnetic radiation to obtain high-quality cross-sectional images of the densely calcified tissues [90]. Methods based on various signal treatment algorithms (UTE, ZTE, SWIFT) have been developed for the visualization of bone or dental tissues in vivo) [91–93]. Studies from Idiyatullin et al. [92, 93] assessed the feasibility of MRI on isolated cracked teeth with a detection limit of around 20 µm, and the work also provided evidence that simultaneous three-dimensional imaging for hard and soft-tissue of teeth can be realized. It should be mentioned that MRI has the potential to image subtle dental structures including microcracks. However, MRI is not commonly applied to cracked tooth diagnosis due to its high cost and operational difficulty [90, 94].

Various effective diagnostic methods currently available for diagnosing cracked tooth are briefly summarized in Table. 2. According to the light sources, they can be divided into three types: X-ray-based method, ultrasound-based method and optical light-based method. Among X-ray-based methods, CBCT is considered one of the primary diagnostic tools for cracked tooth detection. Its overall performance (including the convenience of the operation, the 3D visualization and low-radiation) made it stand out from other ray-based medical imaging techniques (oral radiography and micro-CT). Researchers and engineers have also made some efforts to use the non-invasive ultrasonic detection method. However, the complex mechanism and specialized knowledge may somehow hinder its further applications. The optical light-based method, particularly the OCT technology seems to be maturing because of its distinct advantage of non-radiation and ultra-high resolution (around 10 μm/pixel), but careful image treatment algorithms may be necessary for denoising and crack identification [50].

**Table 2:** Summary of imaging diagnostic techniques for cracked tooth

Method	Voxel size	Width can be detected	Radiation	Advantage	Disadvantage
Oral X-rays			Lower	Wide range of applications, cheap	Low efficiency, anatomic superimposition, distortion
CT			High	Fast, Three-dimensional imaging	Expensive, presence of artifacts, low spatial resolution
CBCT	75–400 μm [51] 125–2000 μm [34] 80 μm [49] 250 μm [50]	50–300 μm [148]	Low	Easily operate, safe, cheap, accurate, High spatial resolution	Difficult to obtain good soft tissue detail, presence of artifacts
Micro-CT	5–20 μm [89] 13.67 μm [67]	5–20 μm [89]	Extremely high	High spatial resolution, fast, and precise	Cannot be applied in vivo
Ultrasound		4–35.5 μm (VibroIR) [78]	No	Non-invasive, painless, accurate, visualization of hard and soft tissue, and good acceptance by patients	Difficult to operate
OCT		10 μm [86]	No	High resolution, non-invasive, cheap, accurate, real-time imaging, safe	Noise in the image
MRI		Around 20 µm [93]	No	Non-invasive, Contrast resolution,	Noisy, expensive, easily distorted by metal

## AI-based image analysis for crack detection

**Fig. 6:**
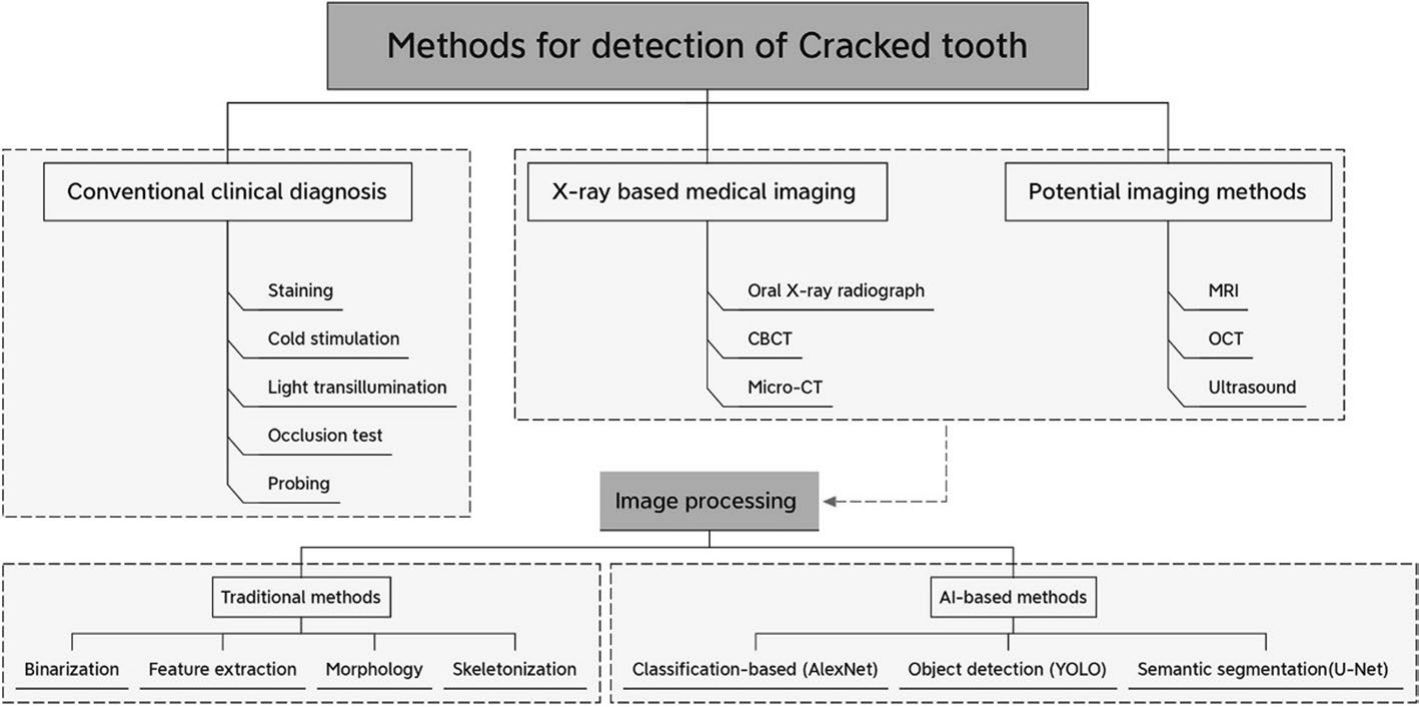
Block diagram to illustrate the framework of the methods for detection of cracked tooth

As summarized in Fig. 6, one of the meaningful and fascinating research directions for the crack diagnosis may be considered as further image treatment or analysis for the related medical images. Images generated from medical diagnostic systems are often accompanied by noise, which may sometimes make the image so blurred that even the experienced doctors may omit or misdiagnose. On the other hand, manual diagnosis based on medical imaging probably requires extensive labor. For some medical devices with high operational requirements, such as MRI and ultrasound, this may also bring some challenges to radiologists, especially the young doctors. Since automation and specialization continue to evolve, the treatments or image-based detection algorithms for the obtained diagnostic images may become one of the main research tasks in the future. Some pioneers have made a few explorations. For example, Kim et al. [53] utilized Hough transformation to realize image denoise and automatic crack detection for OCT images. In addition, automatic segmentations implemented by CNN had been achieved on MRI images of brain tumors, mammograms and radiographs of bone tissue [18, 19, 95]. More recently, Zhang et al. [96] proposed a method based on digital image correlation to identify the crack path and quantitative characterization of crack opening displacement, whose results were in good accordance with the one obtained from micro-CT.

It is a pity that few papers could be found concerning the issue of image treatments aiming for cracked tooth detection, but image-based crack detection has already been widely developed in engineering structural monitoring, including bridges, dams, the wing of the plane and so on. In this section, the currently established methodologies, especially AI-based crack detection algorithms are summarized, which may hopefully provide some valuable suggestions or guidance for further dental crack diagnosis.

**Fig. 7:**
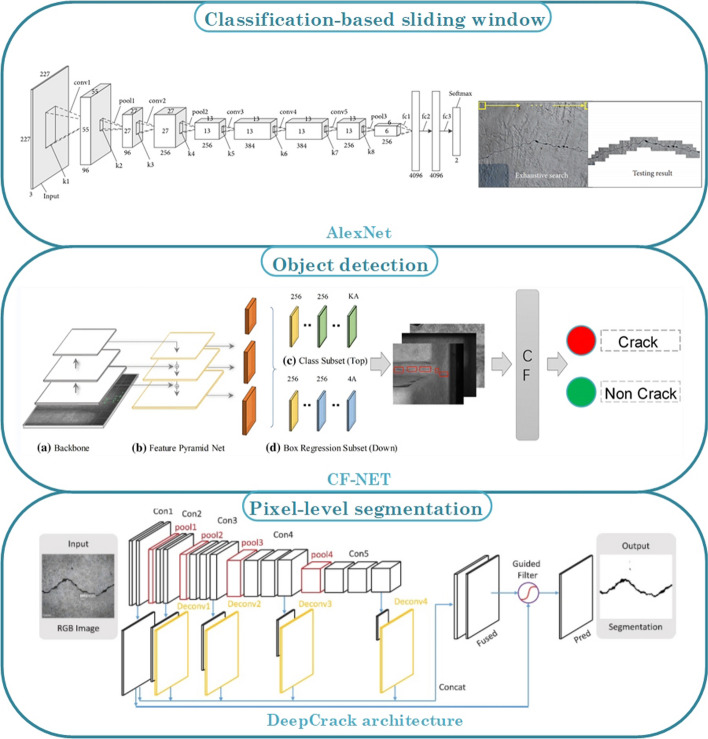
Representative frameworks of convolutional neural network for crack detection and segmentation

Similar to the cracked tooth, tiny cracks may often occur in the engineering structure due to the presence of fatigue stresses and cyclic loads, which could further lead to huge safety disasters [97]. However, manual recognition of a large number of images may be inaccurate due to inspectors' visual fatigue or errors in judgment [98]. Various image processing techniques are applied on the image to detect cracks, including morphological operations [99], wavelets [100], image binarization [97, 101], seed growth [102], digital image correlation [103–107], and edge detectors [108]. Whereas, the robustness and adaptiveness of the traditional image treatment methods were quite limited once the imaging conditions (light changes, surface textures and so on) had been changed [109]. Nowadays, in medical image processing, artificial intelligence (especially deep learning) as one of the emerging technologies has brought great benefits for image-based crack detection [21]. Many methods represented by convolutional neural networks have been applied to surface crack detection, which is comprehensively summarized in Table 3 for the last five years. As illustrated in Fig. 7, depending on the way how these methods address the crack detection, they can be divided into three categories: image classification-based methods, object detection-based methods and semantic segmentation-based methods.

**Table 3:** Summary of convolutional neural network for crack segmentation

Methods	Models	Reference	Size of images	Precision (%)	Recall (%)	F1 (%)	Size of data sets
Image classification	DCNN	[111]	256 × 256	99.09			60,000
		[110]	256 × 256	98			40,000
Object detection	YOLO	[118]	448 × 448	83.54	79.93		2000
	YOLO-v2	[21]	227 × 227	89			990
		[119]	416 × 416	88.51	87.1	87.8	9053
	YOLO-v3	[120]	416 × 416	89.16	91.16		1500
		[121]	480 × 600	88			4000
	Faster R-CNN	[115]	1865 × 2000	78.53	85.56		3000
		[116]		96.3			5966
		[117]	500 × 375	90.2			2366
Semantic segmentation	FCN (VGG19)	[126]	224 × 224	81.7	78.97	79.95	> 800
	FCN (VGG16)	[128]	227 × 227	90		89.3	
	U-Net	[131]	572 × 572	92.46	82.82	87.38	118 (CrackForest)
		[136]	512 × 512	92.12	95.7	93.88	118 (CrackForest)
		[125]	48 × 48	90	91	90	57
		[134]	320 × 320	94.94	93.55	96.37	118 (CrackForest)
		[133]	256 × 256	97.02	94.32	95.55	118 (CrackForest)
		[137]	480 × 320	91.45	88.67	90.04	1200
		[138]		97.31	94.28	95.75	118 (CrackForest)
	CrackU-net	[132]	1024 × 1024	98.56	97.98	98.42	3000
	SegNet	[130]	360 × 480	90.92	97.47	79.16	1021
		[129]	608 × 608	80.31	80.45		504
	CrackSeg	[139]	256 × 256	98	97.85	97.92	8198
	SDDNet	[140]	513 × 513	87.4	87	87.2	537
	FPCNet	[141]	288 × 288	97.48	96.39	96.93	118 (CrackForest)

Image classification-based methods essentially treat the crack detection problem as a binary classification problem. The work from Dorafshan et al. [98] had shown that CNN-based classification methods had a significant advantage both in terms of detection speed and accuracy compared to the common edge detectors (i.e., Roberts, Prewitt, Sobel, Laplacian of Gaussian, Butterworth, and Gaussian). Cha et al. [110] in 2017 proposed DCNN based on the sliding window, which improved the accuracy of crack detection to a level up to 98%. After two years, Li et al. [111] applied classical Alexnet as a classification network, and with the help of the larger data set, the resulting precision metric further improved with a value of around 1.09%. However, traditional CNN with a sliding window needed to check all possible locations within the image, which would decrease the detection efficiency.

Without specifying the size of the filtering window, object detection-based methods directly provided the information of the position and dimension of the targets of interest with a bounding box labeled in the image. The representative networks of the object detection-based methods were reported to be RCNN [112], YOLO [113] and their variants [114]. Two-stage algorithms represented by Faster-RCNN realized high accuracy in object detection, which was reported to be used to overcome the challenging task of crack detection in complex conditions. Ibragimov et al. [115] and Li et al. [116] used Faster-RCNN-based model to achieve fast detection of different types of cracks, such as longitudinal, transverse, alligator and so on. Cha et al. [117] proposed a structural damage detection method based on optimized Faster R-CNN, which can overcome some disadvantages of conventional CNN-based techniques, such as the low-speed testing and the inappropriate sliding window size.

**Fig. 8:**
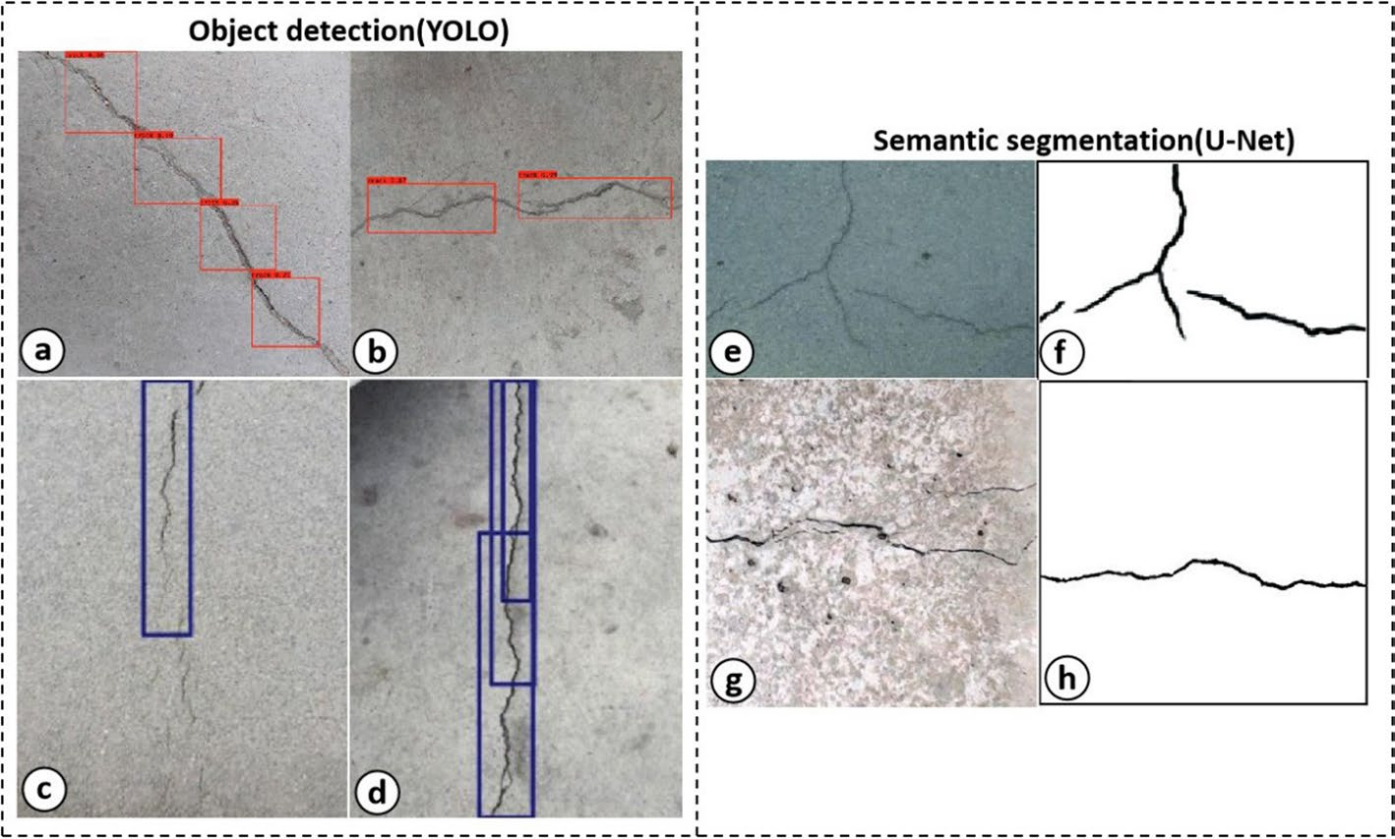
Typical results of object detection (YOLO) [120] and semantic segmentation (U-Net) [136] under different situations. The anchor boxes in the figure (**a**–**d**) represent the area of crack. **a** Testing results of normal crack in the smooth pavement. **b** Testing results of normal crack in the rough ground. **c** Tiny crack in the smooth pavement. **d** Normal crack in the stained ground. For semantic segmentation methods, **e**, **g** represent the original images under shadow and rough pavement, respectively. **f**, **h** Results of semantic segmentation of the crack. Copyright © Elsevier publishing (2019)

The YOLO series, as one of the outstanding representative networks of one-stage object detection algorithms, have dramatically improved the detection speed. Li et al. [118] used the improved YOLO to effectively improve the detection accuracy and real-time detection speed, which solved the difficult problem of locating track plate cracks. Mandal et al. [119] proposed an automated pavement cracks analysis system based on the YOLO-v2, which could detect and classify various kinds of cracks, such as longitudinal, lateral linear and alligator cracks. Teng et al. [21] found that the ‘resnet18’ model as the YOLO_v2 feature extractor in detecting concrete cracks performed best among eleven different CNN models (e.g., ‘squeezenet’, ‘mobilenetv2’, ‘vgg16’, etc.). However, the accuracy of these algorithms is rather limited. To further improve the crack detection speed and accuracy, Zhang et al. [120] proposed a lightweight crack detection algorithm based on YOLO-v3 by combining with MobileNets and convolutional block attention module (CBAM), which successfully achieved higher accuracy and faster detection speed compared to the original one. Nie et al. [121] further improved YOLO-v3 on the multi-scale prediction, basic classification network and classifier, which seemed to take into account for both pression and detective efficiency. Some typical detection results obtained from YOLO-v3 are presented in Fig. 8a–d.

Instead of labeling or bounding boxes for the diagnostic interest, pixel-level crack segmentation methods exhibited great potential for crack detection, which enabled to extract precise information and more detailed features, such as crack path, position, length, width and density [122]. Semantic segmentation, also named semantic segmentation, could be considered as the process of designating each pixel in the input image to its corresponding class [123, 124]. Typical semantic segmentation method used for crack detection was represented by FCN, Seg-Net and U-net [125].

FCN is an end-to-end convolutional network consisting of downsampling and upsampling parts, which can predict and classify each pixel point while retaining the spatial information of the input image. Yang et al. [126] used FCN to detect cracks at the pixel level. Although the accuracy of this model is lower than CrackNet [127], the training time significantly declined to less than 1 h. Dung et al. [128] chose VGG16 as the encoder of the FCN architecture for concrete crack detection and density evaluation, which reached about 90% for both precision and F1-Scores (harmonic mean of the rate between precision and recall).

The Seg-Net model uses a symmetrical structure of encoder and decoder parts, which is conducive to preserving detailed features. Feng et al. [129] proposed a SegNet-based network to detect crack on the dam surface showing higher indicators and performance compared with other pixel-level detection networks, such as FCN, U-Net, ResNet152-based and SegNet. Jang et al. [130] used a ring-type climbing robot based on a modified SegNet for the crack detection of a high-rise bridge pier, which can overcome the limitations of manual inspection, such as time-consuming, inaccessible area and false judgment.

As one of the modified fully convolutional neural network models, U-Net was initially proposed in medical image processing [20]. Many applications using U-net could be found, i.e., segmentation of cells or bacteria in microscopic images. In recent years, U-Net has been widely applied to crack detection, especially in the engineering field. Jenkins et al. [131] used U-Net architecture to realize automated semantic segmentation for road and pavement surface cracks. However, the detection accuracy is influenced due to some interferences in images, such as noise, irregular patterns, illumination variation and so on. Some researchers began to modify the U-Net to improve the accuracy and robustness. Huyan et al. [132] proposed specific CrackU-net architecture with fixed convolutional kernel size and max-pooling operation, which remarkably showed better performance compared to the FCN and U-Net. Modification of the encoder part of U-Net with a pre-trained ResNet-34 neural network also indicated some improvement in the performance of F1-Score and precision [133]. The intensive task of manual crack image annotation was a troublesome problem. In that scenario, Liu et al. [125] used the U-Net-based segmentation method to identify the crack under different conditions (e.g., lighting condition, rough background, crack width), which achieved higher accuracy with a smaller data set (57 images) compared with the previous FCNs proposed by Yang et al. [126] (> 800 images) and Dung et al. [128] (500 images). Konig et al. [134] modified the U-Net by adding attention mechanism and residual convolutional blocks, which also showed outstanding performance with a small data set (117 images). When dealing with images with the larger size, another U-Net-based architecture combined with sliding window techniques was proposed by Ji et al. [135]. Although it achieved higher accuracy than the Canny edge detector and Sobel edge detector in many complex environments (with stains, pits and scratches), the sliding window-based methods may increase the training and testing time. To solve that, Cheng et al. [136] proposed a novel U-Net-based method to directly generate a crack segmentation from a whole image without splitting the image into small pieces (accuracy above 92%). Some detection results of U-Net are presented in Fig. 8e–h.

To verify whether the model architecture with a larger amount of the hidden layers can obtain better detection accuracy, Zhang et al. [137] analyzed the effect of network depth on four U-Net-based architectures with different numbers of convolution layers (CrackUnet7, CrackUnet11, CrackUnet15, and CrackUnet19). The results showed that the performance of CrackUnet15 and CrackUnet19 was comparable. However, the parameters of CrackUnet19 are significantly large, which requires much more training time than the others. A similar study had also been conducted by Escalona et al. [138] (U-Net based network equipped with different convolutional layers (U-Net-A (23 layers), U-Net-B (11 layers), U-Net-C (7 layers)), and the results indicated that the U-Net-B with 11 layers might be a proper choice for the hidden layers due to its highest accuracy and shortest training time. Therefore, a profound consideration of the network architecture especially the number of the convolutional layers would be crucial to both ensure the detection accuracy and the detection speed. Furthermore, by introducing the multiscale dilated convolution module and fusing the high spatial resolution features of the shallow network in upsampling module process, Song et al. [139] presented the so-called CrackSeg, which possessed remarkable feature extraction ability on complex backgrounds. Particularly, the network (SDDNet) presented by Choi et al. [140] consisted of various multi-functional modules significantly improve the anti-interference capability and the detection speed. Besides, network-FPCNet proposed by Liu et al. [141] showed a certain degree of improvement for the detection speed and accuracy due to the modification of the upsampling process and the addition of the multi-dilation module.

## Conclusions and future perspective

The diagnosis of cracked tooth has experienced from traditional clinical treatments to medical imaging methods. In this review, various medical imaging technologies have been summarized. Among the X-ray-based methods, oral X-ray radiograph can effectively diagnose common cracked tooth with extremely low radiation dose, but the anatomic superimposition in 2D oral X-ray radiograph may influence the diagnostic accuracy. CBCT, as a three-dimensional imaging diagnostic system, plays a crucial role in the diagnosis and therapeutic evaluation of cracked tooth. As for micro-CT, although it has the highest spatial resolution, it is improper to be directly applied in clinical diagnosis due to its extensive radiation doses. Other emerging medical imaging techniques such as ultrasound and MRI were also investigated due to the non-radiative and non-invasive characteristics. However, these modalities are currently only tested on isolated or simulated teeth, and the complex interaction mechanism and specialized knowledge may somehow hinder its further applications in the clinic. Particularly, OCT as an emerging technology has been considered as one of the auxiliary diagnostic tools for the detection of the cracked tooth due to its characteristics of high resolution, non-invasive, accurate, and real-time imaging. As indicated by some researchers, to further increase the accuracy, image treatment algorithms on medical diagnostic images (such as CBCT, OCT, etc.) may be one of the key research directions for the diagnosis of the cracked tooth.

In recent years, AI with deep learning as the core has developed rapidly, some outstanding CNN-based algorithms have dramatically improved the efficiency and accuracy in aid of the diagnosis of various medical problems. Similar to cracked tooth, tiny cracks as a kind of damage existed extensively in engineering. Three types of CNN-based crack detection methods (image classification, object detection, semantic segmentation) are comprehensively overviewed. To be more specific, image classification-based algorithms (Alexnet) essentially treated the crack detection problem as a binary classification problem. However, its efficiency was somewhat limited due to the sliding window-based algorithm. Object detection-based algorithms (YOLO, Faster R-CNN) directly provided the information of the position and dimension of the targets of interest with a bounding box labeled in the image. Pixel-level crack segmentation algorithms (Unet, Segnet, CrackSeg) exhibited great potentials for crack detection, because they cannot only provide the location of the crack, but also extract precise information and more detailed features, such as crack path, position, length, width and density.

It should be pointed out that image-based intelligent auxiliary diagnosis may be one of the primary directions in clinical applications. Although image processing is currently less used in the diagnosis of cracked tooth, there is a reasonable prospect that AI-based diagnostic workflow would occupy an important place in clinical dental diagnosis. Compared to the traditional imaging workflow that heavily relies on human labor, AI enables more safe, accurate and efficient imaging solutions [142]. However, a number of technical issues may still exist in AI-based detection, such as heavy computational cost, issues of the selection for optimal parameters and formation or pre-processing of the training data sets. In this review, the currently established methodologies, especially AI-based crack detection algorithms used in engineering structure, were comprehensively reviewed, which may hopefully provide additional valuable suggestions or guidance for further dental crack diagnosis. In perspective, the AI-based detection methods were suggested to be combined with various imaging modalities (OCT, CBCT, etc.), which may provide the worthy or amazing diagnostic methods with more intelligent, automated and specialized solutions.

In addition to medical imaging analysis, some applications could be further realized for other related thermographic fault diagnosis in engineering. Recently, Adam Glowacz innovatively proposed the methods of feature extraction and fusion for thermal imaging (namely, Binarized Common Areas of Maximum Image Differences—Fusion method [143] and Common Part of Arithmetic Mean of Thermographic Images method [144]), which was demonstrated to be quite efficient for fault diagnosis of electrical devices and electric power tools. Similar to medical imaging, thermal images may present diverse features between machines with and without faults. AI-based image treatment algorithms, particularly the CNN, can automatically learn the features after well trained, which could be used to distinguish different types of faults according to the temperature information of abnormal areas, thereby realizing intelligent recognition and automatic diagnosis.

## Data Availability

Not applicable.

## Abbreviations

CNN: Convolutional neural network
AI: Artificial intelligence
CT: Computer tomography
CBCT: Cone-beam computed tomography
Micro-CT: Micro-computed tomography
OCT: Optical coherence tomography
MRI: Magnetic resonance imaging
VRF: Vertical root fractures
VibroIR: Vibrothermography
CBAM: Convolutional block attention module
PR: Periapical radiography
SS-OCT: Swept-source optical coherence tomography
PPV: Positive predictive value
NPV: Negative predictive value
SEN: Sensitivity
SPE: Specificity
ACC: Accuracy
